# Impact of a comprehensive review template on personalised care in general practice for patients with multiple long-term conditions: a mixed-methods evaluation

**DOI:** 10.3399/BJGPO.2025.0022

**Published:** 2025-12-19

**Authors:** Caroline Coope, Dereth Baker, Kate Alice Lippiett, Alice Moult, Lauren J Scott, Simon Chilcott, Andrew Turner, Clare Jinks, Maria Carmen Portillo-Vega, Krysia Dziedzic, Cindy Mann, Richard Byng, Grace Scrimgeour, Chris Salisbury, Rachel Johnson

**Affiliations:** 1 Centre for Academic Primary Care, Bristol Medical School, University of Bristol, Bristol, UK; 2 School of Medicine, Keele University, Staffordshire, UK; 3 NIHR ARC Wessex, School of Health Sciences, University of Southampton, Southampton, UK; 4 NIHR ARC West, Bristol Medical School, University of Bristol, Bristol, UK; 5 Public Contributor, University of Bristol, Bristol, UK; 6 Peninsula Medical School, University of Plymouth, Plymouth, UK

**Keywords:** multimorbidity, annual review, organisation of care, primary health care, general practice

## Abstract

**Background:**

Primary care is in urgent need of more effective and efficient ways of managing the care of people living with multiple long-term conditions (MLTCs; multimorbidity). Personalised care organised around an individual’s needs and conditions, taking account of individual context and priorities and supporting self-management, may offer an improved approach.

**Aim:**

To explore the impact of a computerised template to support personalised care for patients with MLTCs within the context of routinely applied general practice.

**Design & setting:**

A convergent mixed-methods evaluation design. General practices were recruited from three areas of England: Bristol, Southampton, and Staffordshire.

**Method:**

A computerised template for the review of MLTCs was made available to all general practices subscribing to a commercial template supplier. Implementation practices were supported to conduct personalised multimorbidity reviews. We used routine clinical data from implementation and control practices, a before-and-after patient questionnaire, and qualitative interviews with general practice staff and patients to evaluate the impact of the intervention.

**Results:**

Thirty-two general practices were recruited, of which half were implementation practices. Using the multimorbidity template has potential to improve quality of care and patient benefit with no increase in consultation numbers. Patients received a more complete assessment of their needs with a clearer focus on the problems that matter most to them. Conducting multimorbidity reviews can increase burden on nursing staff and consideration is required as to the organisation of reviews and appropriate training for nursing staff.

**Conclusion:**

Use of the multimorbidity template needs to be supported by staff training, adequate practice capacity, support for system reorganisation, and attention to incentives to facilitate its benefits.

## How this fits in

There is a need to improve personalised care for people with multiple long-term conditions (MLTCs), but trials of interventions have shown limited benefits and problems with implementation, partly owing to the constraints of a randomised trial. We used a mixed-methods pragmatic approach to evaluate the impact of a computerised template to support personalised annual review of patients with MLTCs when implemented as routine care in real-world general practice. We found that this approach has potential to improve the quality of care for patients while offering efficiencies to the practice. However, challenges to implementation remained owing to a lack of generalist skills in staff members who conduct annual reviews, largely practice nurses.

## Introduction

More than one-quarter of the UK population have two or more long-term medical conditions (multimorbidity).^1^ Managing the growing numbers of people living with multiple long-term conditions (MLTCs) poses a major challenge for health services.^2,3^ With the UK prevalence of people with four or more long-term conditions (LTCs) set to increase from 9.8% (2015) to 17% by 2035,^2^ health service demand will increase, particularly in general practice, the first point of contact for most LTC care.^4^ Patients with MLTCs have a higher number of general practice contacts per year than patients with one or no chronic diseases.^5^

Alongside this increase in the prevalence of MLTCs runs a health system predicated on offering services for single diseases. In England, annual condition-specific health reviews facilitate the monitoring of patients’ LTCs within primary care, which is incentivised within the pay-for-performance Quality Outcomes Framework (QOF).^6^ Annual LTC reviews have generally been conducted by a member of the general practice team and guided by evidence-based protocols of best practice, often facilitated via computer templates.^7^ However, for the management of patients with MLTCs, this is recognised as inefficient and time-consuming for staff and patients owing to duplication of work and repeated visits to the general practice.^8,9^

Finding more effective and efficient approaches to organising primary health care for patients with MLTCs is a healthcare and research priority.^10^ One proposed approach is that of personalised care (also referred to as person-centred care), comprising care organised around the person’s needs and conditions, taking account of individual context and priorities, and supporting self-management.^11,12^ Evidence suggests that personalised care planning for the management of single LTCs can result in improvements in patient outcomes,^13^ although evidence of the impact of this approach for patients with MLTCs is limited. One trial that compared routine practice with a person-centred care model for patients with MLTCs showed improvements in patients’ experience of care but not in health-related outcomes.^14^ This trial identified challenges associated with insufficient staff training and suboptimal implementation of the intervention in the context of a randomised trial.^15^

In this study, we aimed to explore the impact of a commercial computerised template to support personalised care for patients with MLTCs within the context of routine general practice. Specifically, we explore whether use of the template for multimorbidity reviews supports personalised care and facilitates service benefits (workload, clinical quality, efficiency), staff benefits (job satisfaction, confidence in delivering multiple-condition care), and patient benefits (improved personalised care and reduced treatment burden).

## Method

### Research design

A convergent mixed-methods design was used. Qualitative and quantitative data were collected in parallel, analysed separately, and the results were integrated.^16,17^ The Good Reporting of A Mixed Methods Study (GRAMMS) guideline is used to report the study (see Supplementary Table S1).^18^ Details of study design are presented for each type of data at the beginning of their headed section.

### The intervention

The computerised template was made available to all general practices in England subscribing to Ardens (a commercial supplier of templates) in 2022. Before this research, Ardens had introduced a multimorbidity template designed to fulfil QOF requirements for common LTCs. The study team provided feedback to Ardens, building on the team’s previous work,^19^ with a view to incorporating elements fundamental to personalised care, such as an initial question asking what matters most to the patient, questions about wellbeing, pain and mental health, links to social prescribing, and a care and support plan based on patient priorities. Additionally, the layout was redesigned to reduce duplication and support a streamlined consultation in which only sections relevant to an individual’s LTCs were visible during the review.

### Implementation approach

General practices were recruited into the study (implementation practices) and supported by the local research team to plan an approach to conducting multimorbidity reviews tailored to their general practice needs and capacity. There was an intended two-part review process mirroring the multimorbidity template design. First, an initial consultation (IC) is conducted comprising clinical tests and measures (for example, weight and blood pressure measurement). This is followed by the main annual review (AR) consultation envisaged to occur at a separate appointment to allow all IC test results to be available and populated in the main template for discussion. Before the AR, practices were encouraged to send patients additional preparation materials developed by Year of Care (YoC) Partnerships^20^ that aimed to promote patient involvement in care. The YoC patient preparation materials incorporate the pre-populated test results completed at the IC, along with prompts to help patients think about questions or concerns they might wish to discuss at their AR consultation. It was envisaged that the IC could be conducted by a healthcare assistant (HCA) while the AR would need to be conducted by a trained nurse or GP, depending on the complexity of patient needs and the capacity of the practice team.

### Logic model

**Figure 1.**
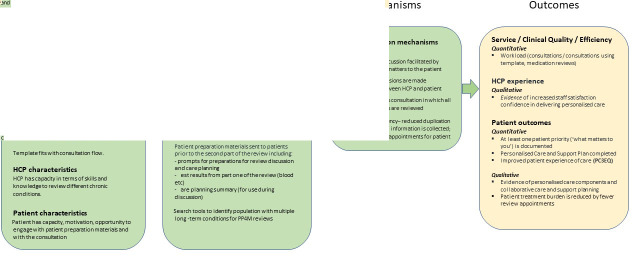
Logic model to illustrate how the intervention was theorised to influence outcomes. HCP = healthcare professional. PC3EQ = Person-Centred Coordinated Care Experience Questionnaire. PP4M = Personalised Primary care for Patients with Multimorbidity. QOF = Quality and Outcomes Framework.

A logic model was developed to support implementation and to illustrate how the intervention was theorised to influence outcomes, with the latter only presented here (Figure 1). The outcomes were used to inform the mixed-methods analysis approach.

### Study setting and practice eligibility

The study was conducted in three areas of England: Bristol, Southampton, and Staffordshire. General practices needed to be using the EMIS computer system, have a subscription to Ardens templates, and have a practice list size of at least 5000 patients to ensure enough eligible patients. We recruited control practices similar to implementation practices in terms of region, deprivation, and list size.

### Quantitative study design

The study involved a non-randomised controlled before-and-after study using routine clinical patient data from implementation and control practices and a before-and-after questionnaire of patients experiencing the intervention in implementation practices.

Patients were eligible for inclusion if they met the inclusion criteria (Table 1) at all three data extraction timepoints of January 2021, April 2022, and July 2023.

**Table 1.** Patient inclusion and exclusion criteria

Inclusion criteria: Registered with a practice that agrees to take part in the study Aged ≥18 years Have at least three types of chronic health condition, some of which were grouped (see Supplementary Box 1: Long-term condition counts), including at least one which is already subject to annual review Due to be invited by their practice for an annual review of their chronic conditions at the practice within 12 months from the start of the study
Exclusion criteria: Patients not invited for annual review under normal circumstances, for example, if they have a terminal illness Some patients who are housebound or in a nursing home, if it is not possible to conduct the same type of ‘template-based’ review away from the surgery

### Recruitment

All general practice in the three regions that met the eligibility criteria were invited by their local clinical research network to express an interest in the study. Interested general practices were then sent detailed information and those still interested were followed up and recruited. We particularly focused recruitment efforts on practices representing deprived areas. Following recruitment of implementation sites, we invited practices that had initially expressed interest in the study to take part as control practices, and then recruited additional practices to ensure that implementation and control practices were similar in terms of deprivation and practice list size.

### Routine clinical data

#### Sampling and data collection

Data were extracted from anonymised electronic medical records for all eligible patients from implementation and control practices to include a pre-implementation period (1 January 2021–31 March 2022) and an implementation period (1 April 2022–30 June 2023).

Data extracted included the following: patient characteristics (number and type of LTCs [grouped], age in 5-year age groups, sex [male or female], ethnicity, and Index of Multiple Deprivation [IMD] quintiles); process and outcome variables (patients invited for an MLTC review, attended for MLTC review, completion of different components of the multimorbidity template including review of specific diseases); practice workload (frequency and type of consultation and staff group who performed the consultation)*;* personalised care and support plan (offered, agreed, reviewed, and given to patient). Details of variable measures can be found in Supplementary File 1.

#### Analysis of routine data

All eligible patients in implementation and control practices were compared for the pre-implementation and implementation periods. Descriptive analysis included the summary of continuous variables using means and standard deviations (SD), or medians and interquartile ranges (IQR) if the distribution was highly skewed. Categorical data were summarised as numbers and percentages.

Comparative analyses were conducted using the routine data for the number of consultations by different types of staff (workload), whether patients with specific major health conditions had been reviewed for that condition (clinical quality) and whether a personalised care and support plan had been offered, agreed, reviewed or given to the patient (a key measure of personalised care, reported under patient benefits). The number of consultations (workload) was explored using mixed effect negative binomial regression at the practice level. Whether patients were reviewed and whether care plans were offered or provided were analysed using mixed effect logistic regression models. In both cases, a difference-in-difference approach was used, presenting results using incident rate ratios or odds ratios for the interaction term (that is, the additional effect of the intervention in the implementation period, over and above any effect of period and/or implementation group alone).

### Patient questionnaire

#### Sampling and data collection

Implementation practices were asked to send the Person-Centred Coordinated Care Experience Questionnaire (P3CEQ)^21^ to patients invited to attend their AR consultation. Patients who completed a questionnaire and had been reviewed were invited to complete a follow-up questionnaire about 4 weeks after their review.

#### Analysis of questionnaire data

Details of scoring patient questionnaires can be found in Supplementary File 1. The analyses compared scores (as a continuous measure) pre- and post- the MLTC review. Mixed effects linear regression was used to compare the mean score at the two timepoints. A pre- and post- indicator was fitted as a fixed effect and patient as a random effect. Mean differences and corresponding confidence intervals (CIs) and *P* values are presented. Quantitative analysis was conducted using Stata (version 17.0).

### Qualitative

#### Sampling and data collection

Implementation practices chose whether or not to take part in the qualitative study.

*Semi-structured interviews with patients:* healthcare staff invited patients attending for MLTC reviews to an interview, excluding those lacking capacity to consent or assessed as inappropriate to interview. We used maximum variation sampling of patients in relation to known patient characteristics, including MLTCs, where possible.

*Semi-structured interviews with primary care staff:* a nominated contact in a practice invited healthcare professionals involved in using the multimorbidity template, and administrative staff involved in organising MLTC reviews, to interview. Primary care staff were sampled purposively, using maximum variation (different practices and professional groups).

Researchers developed topic guides informed by relevant theory or frameworks (normalisation process theory for implementation evaluation and NHS personalised care framework) to support semi-structured interviews with patients and primary care staff.^11,22^

### Data analysis

An abductive thematic codebook analysis approach was used for the analysis of patient and staff interviews, supported by the personalised care framework,^23^ as shown in Supplementary File 1. An abductive approach supports analysis that moves iteratively and recursively between an initial theoretical framework and accumulated empirical and theoretical materials.^24^

### Patient and public involvement

A patient and public involvement (PPI) group were involved in the study development and data analysis processes. The details have been published elsewhere.^25,26^

### Mixed methods: integration of quantitative and qualitative data

Integration of the quantitative and qualitative results takes place in the presentation of results together under joint themes and in the discussion.

## Results

The results are presented first to show characteristics of participating practices, patients, and staff, then to demonstrate the extent of implementation (to aid interpretation of the outcome results), and then in line with the outcomes section of the Figure 1 logic model, under the headings of service benefits (which relates to services, clinical quality, and efficiency), staff benefits (which relates to healthcare professional experiences), and patient benefits (which relates to patient outcomes). Selected verbatim quotations from participants are presented in the text, with supplementary quotations referred to in Supplementary Table S4 to evidence trustworthiness of the results.

### Implementation and control practice sample

Thirty-eight practices expressed an interest in taking part as implementation practices. We recruited 16 implementation practices and the same number of control practices from three integrated care boards (ICBs) regions (Table 2). More than half of the practices were in the 40% most deprived areas (IMD quintiles 1 and 2). Of the 16 implementation practices recruited, two did not implement the intervention during the study period.

**Table 2.** Description of study implementation and control general practices

	General practices			
**Characteristic**	Implementation *n* = 16^a^		Control *n* = 16	
Integrated care board region	*n*	%	*n*	%
A	9	56	9	56
B	4	25	4	25
C	3	19	3	19
List size^b^				
Small	5	31	3	19
Medium	8	50	9	56
Large	3	19	4	25
IMD quintile^c^				
1	4	25	6	38
2	5	31	3	19
3	3	19	1	6
4	2	13	1	6
5	2	13	5	31

^a^Two practices did not implement the intervention.^b^Small <10 000; medium 10 000–20 000; large >20 000. ^c^1 = most deprived. IMD = Index of Multiple Deprivation

### Patient cohort comparing implementation and control practices

Similar numbers of eligible patients were identified in the implementation (*n* = 5060) and control practices (*n* = 5363) (Supplementary Table S2). Patient cohorts were well matched across a range of characteristics (age group, sex, IMD, number of LTCs), except that control practices had a higher proportion of patients from minority ethnic groups (3.0% versus 8.3%).

### Patient and staff qualitative interview sample

Staff interviewed (*n* = 33) included healthcare assistants (HCAs), nurses, GPs, and administrative and management staff (Supplementary Table S3). Patients interviewed (*n* = 21; aged 37–92 years) represented experiences from seven general practices.

### Extent of implementation

In implementation practices the proportion of eligible patients in whom the multimorbidity template was used increased from 21.6% (*n* = 1095/5060) in the pre-implementation period to 46.1% (*n* = 2331/5060) in the implementation period, compared with 10.6% (*n* = 571/5363) and 15.6% (*n* = 838/5363), respectively, in control practices (odds ratio 2.86 [95% CI = 2.34 to 3.49], *P*<0.001.

### Service benefits: workload, efficiencies, and quality of care

#### Practice workload

Patients in the eligible cohort had a high number of consultations (of all types) in both the implementation and control practices (median 16 consultations per annum). There was no statistically significant difference between implementation and control practices in the post-implementation compared with pre-implementation period in respect to number of consultations, type of consultation, or practitioner profession (Table 3).

**Table 3.** Comparison of total number of general consultations (practice workload)**,** pre- and implementation period by implementation and control practices

	Intervention practices				Control practices					
	Pre-intervention period (*n* = 5060)		Intervention period (*n* = 5060)		Pre-intervention period (*n* = 5363)		Intervention period (*n* = 5363)		Incident rate ratio* (95% CI)	*P* value
	Med	IQR	Med	IQR	Med	IQR	Med	IQR		
All eligible patients:										
Number of consultations	16	(9, 25)	16	(9, 27)	15	(9, 25)	16	(9, 25)	1.04 (0.95 to 1.13)	0.425
By consultation type										
Face-to-face/home	7	(4, 13)	9	(5, 15)	7	(4, 12)	9	(5, 15)	1.01 (0.90 to 1.14)	0.845
Telephone, video, or e-consult	7	(3, 13)	6	(3, 12)	8	(4, 13)	6	(3, 10)	1.09 (0.91 to 1.31)	0.335
By practitioner										
GP	5	(2, 11)	5	(2, 11)	6	(2, 11)	6	(2, 11)	1.07 (0.98 to 1.18)	0.132
Nurse or paramedic	5	(2, 9)	5	(2, 9)	5	(2, 9)	4	(2, 8)	1.07 (0.93 to 1.24)	0.336
HCA	2	(1, 4)	2	(1, 5)	1	(0, 3)	1	(0, 3)	1.09 (0.82 to 1.45)	0.541
Pharmacist or pharmacy technician	0	(0, 1)	0	(0, 1)	0	(0, 2)	1	(0, 2)	0.82 (0.50 to 1.36)	0.447
Other	0	(0, 1)	0	(0, 1)	0	(0, 1)	0	(0, 2)	1.13 (0.79 to 1.63)	0.500

^a^The presented incident rate ratios are the interaction term between timeperiod and implementation or control group from the difference-in-difference model; that is, the additional effect of the implementation in the post-implementation period, having adjusted for the effect of timeperiod and implementation or control group. Variables for mean age, mean Index of Multiple Deprivation decile, percentage White and percentage female per practice, were fitted as fixed effects. Practice ID was fitted as a random effect, with practice size fitted as the offset. Counts for each consultation type and practitioner were calculated (and analysed) separately.

HCA = healthcare assistant. Impl = implementation. IQR = interquartile range. Med = median.

#### Staff workload

Some nurses found conducting the multimorbidity reviews more demanding than single disease reviews owing to their longer length and intensity (typically 20–45 minutes). Albeit many nurses believed that, in the long run, multimorbidity reviews might offer opportunities to decrease workload:

*'They [multimorbidity reviews] are very tiring … I just had a whole day and I think I had 10 back-to-back ‘cause it was easier to do that than it was to do anything else. But by the end of the day I was a bit frazzled.'* (H51_P33, nurse, F)

*'It takes longer [multimorbidity review] but … it probably equates to less appointments when you consider what they’re having through the year [single disease reviews].'* (H42_P6, nurse, F)

Additional work from reviewing several diseases included needing to make multiple referrals (Supplementary Table S2_01). Staff working in deprived areas felt their workload was higher, compared with less deprived areas, in part owing to difficulties engaging their patient cohort (Supplementary Table S4_02).

#### Potential efficiencies

GPs and nurses felt that the multimorbidity template offered the potential to reduce fragmentation of care (Supplementary Table S4_03). They reported the multimorbidity template improved collection of QOF information and other incentivised health targets needed for financial payments (Supplementary Table S4_04):

*'… we pick up more QOF points and anything else that we want to add in, like we recently had the weight management incentive, we do their height and everything anyway so it was the ideal time to just go "do you want a referral?" at the same time, minimum effort but an added income.'* (H42_P6, nurse, F)

#### Quality of care

The proportion of patients with specific conditions who received a review of that condition increased during the implementation period in both implementation and control practices compared with the pre-implementation period (Table 4). This increase was greater in the implementation practices compared with controls for several conditions including diabetes, coronary heart disease (CHD), stroke, atrial fibrillation, chronic obstructive pulmonary disease (COPD), and mental health problems, but was greater in the control practices for rheumatoid arthritis.

**Table 4.** Mixed effect logistic regression model to show the proportion of patients with specific conditions, which had a review of that condition in the pre- and implementation periods by implementation and control practices

	Implementation practices				Control practices					
	**Pre-period** **(*n* = 5060)**		**Impl-period** **(*n* = 5060)**		**Pre-period** **(*n* = 5363)**		**Impl-period** **(*n* = 5363)**		**Odds ratio^a^ (95% CI)**	***P* value**
	***n***	**%**	***n***	**%**	***n***	**%**	***n***	**%**		
**All eligible patients:**										
Dementia	223/414	53.9%	313/414	75.6%	249/461	54.0%	344/461	74.6%	1.05 (0.64 to 1.72)	0.850
Diabetes	2019/3176	63.6%	2448/3176	77.1%	1756/3421	51.3%	2126/3421	62.1%	1.21 (1.01 to 1.44)	0.039
CHD	348/1225	28.4%	522/1225	42.6%	192/1298	14.8%	267/1298	20.6%	1.53 (1.09 to 2.14)	0.015
Heart failure	188/614	30.6%	424/614	69.1%	196/671	29.2%	494/671	73.6%	0.91 (0.59 to 1.38)	0.647
Stroke	305/1470	20.7%	577/1470	39.3%	165/1584	10.4%	299/1584	18.9%	1.86 (1.29 to 2. 69)	0.001
Atrial fibrillation	161/872	18.5%	352/872	40.4%	108/869	12.4%	175/869	20.1%	2.75 (1.73 to 4.38)	<0.001
COPD	884/1332	66.4%	1082/1332	81.2%	1083/1455	74.4%	1174/1455	80.7%	1.84 (1.36 to 2.48)	<0.001
Asthma	1174/1714	68.5%	1268/1714	74.0%	1238/1858	66.6%	1315/1858	70.8%	1.09 (0.86 to 1.38)	0.495
Mental health problems	226/2873	7.9%	285/2873	9.9%	195/2994	6.5%	180/2994	6.0%	1.88 (1.29 to 2.76)	0.001
Rheumatoid arthritis	259/416	62.3%	320/416	76.9%	217/425	51.1%	350/425	82.4%	0.33 (0.19 to 0.56)	<0.001
Learning disability	156/172	90.7%	152/172	88.4%	182/196	92.9%	190/196	96.9%	0.24 (0.06 to 1.01)	0.051

^a^The presented odds ratios are the interaction term between timeperiod and implementation or control group from the difference-in-difference model; that is, the additional effect of the intervention in the post-implementation period, having adjusted for the effect of timeperiod and implementation or control group. Models included age group, sex, ethnicity, and Index of Multiple Deprivation quintile as fixed effects, and patient ID nested within practice ID as random effects. N.B. denominators are patients who have a diagnosis for the specific disease.

CHD = coronary heart disease. COPD = chronic obstructive pulmonary disease. Impl = implementation.

Staff perceived that implementing multimorbidity reviews increased quality of care. GPs felt that the multimorbidity template collected clinically important information (Supplementary Table S4_05), could be used by a wider range of staff, and did not omit important data. Some nurses felt the multimorbidity template lacked the detail of condition-specific review templates:

*'So we found that it was far more applicable to a multitude of staff rather than single templates, which can be quite missing of some of the data you might need.'* (H29_P15, GP, F)

Use of the multimorbidity template enabled a more complete picture of patients’ needs at their annual review helping to ensure LTCs were not left unreviewed, as previously happened (Supplementary Table S4_06 to _08):

Healthcare professionals conducting the main consultation felt it offered the opportunity to focus on what mattered to the patient; this facilitated the identification of important issues that might have been missed (for example, social isolation). Separation of tests and clinical measures from the main consultation was thought to have facilitated a more person-centred approach:

*'One chap … wasn't managing at home so he wanted to be signposted towards getting some help at home and then there’s another chap who was saying he was quite lonely and he was signposted to social prescribers … in a normal consultation, you may not have got that information because you would be doing blood pressure, …. it opened different avenues maybe in conversations compared to a normal conversation you would have during a consultation.'* (H22_P27, nurse, F)

However, the intensity and length of multimorbidity reviews risked fatigue in both clinician and patient and could impact detrimentally on care quality (Supplementary Table S4_09). Having practical tasks in the consultation was felt to dilute the intensity of these reviews.

### Staff benefits

Nurses conducted LTC reviews in most general practices. Nurses, doctors, and managers expressed concerns that nurses may not have adequate knowledge and skills across a range of LTCs to conduct multimorbidity reviews competently and confidently, may feel unable to cope with conditions outside their specialist disease-specific expertise (Supplementary Table S4_10), and may risk mistakes when dealing with complex and interacting symptoms. Where nurses were experienced, multimorbidity reviews were positively assessed:

*'… if somebody is inexperienced and they are pressured into doing it, I think that’s when the mistakes can happen but … where you've got somebody that’s experienced, that can look at that person in a different manner to somebody that is just focussing on … one thing, I think it’s a brilliant idea.'* (H29_P16, nurse, F)

*'… there’s also quite a lot you need to do and I think that’s what often causes the overwhelm … is the breathlessness due to the heart failure and I often see a lot of junior sort of staff members like nurses and doctors struggling with that aspect of it is what exactly is the symptom due to …. so I think definitely a multimorbidity type template is more helpful for a more experienced clinician.'* (H31_P5, manager, F)

### Patient benefits

#### Person-centred coordinated care

Almost half of eligible patients in the implementation practices (46.1%, *n* = 2331) received a multimorbidity review. Of those, 247 returned a baseline patient questionnaire (P3CEQ), of whom 117 completed a follow-up P3CEQ questionnaire. There was a small, statistically significant improvement in the PC3EQ total score and the care coordination subscale, but no significant change in the person-centred subscale (Table 5).

**Table 5.** Person-Centred Coordinated Care Experience Questionnaire (P3CEQ)

P3CEQ survey total and sub-scale scores	Baseline (*n* = 117)^a^	Follow-up (*n* = 117)^a^		
	**Mean (SD)**	**Mean (SD)**	**Mean difference (95% CI)**	***P* value**
P3CEQ total score	17.9 (6.4)	19.1 (6.4)	1.31 (0.31 to 2.30)	0.010
Person-centred subscale	15.8 (5.6)	16.6 (5.4)	0.81 (-0.08 to 1.69)	0.075
Care coordination scale	7.4 (3.6)	8.2 (3.8)	0.83 (0.21 to 1.45)	0.009

^a^Comparison includes patients who provided at least one response to both baseline and follow-up data

The separation of the review into two parts was felt to offer time and space for personalised care. Nurses felt able to focus more on the patient because of the improved flow and structure of the consultation:

*'It was a way of giving time to the nurse or the pharmacist to be able to go through multiple things by having the physical parameters measured in a different appointment. They actually found it much more helpful because a person is a whole, they’re not five different conditions. And personally I found it a good way of kind of teaching them [nurses] how things interact with each other.'* (H29_P15, GP, F)

*'… I feel that it just gives me a better structure with my consultation really. I feel like I’m giving the patient more attention in some ways because I’m not having to jump around as much on the computer.'* (H32_P11, nurse, F)

Having the opportunity to listen to the patient’s concerns and priorities was felt to impact the patient’s capacity for behaviour change and self-care (Supplementary Table S4_12) and to enhance opportunities for identifying needs and offering support (Supplementary Table S4_13). Patients valued being asked about their concerns and felt this enhanced the patient–professional relationship:

*'She involves you in it … she’ll say, "Have you got any concerns?", and she makes it clear if you want to ask questions about anything, then you’re free to do so, you know … You feel that you are being spoken to one-to-one, like, and it is second to none.'* (H26_P69, 72 years, M)

Conversely, some staff felt that asking patients what mattered to them raised issues that they were unable to address because of time or because it was outside of their remit (Supplementary Table S4_11, S4_14).

### Care and support planning

#### Awareness and use of the care and support section of the template

Care planning in implementation practices more than doubled in the post-implementation period compared with pre-implementation across patients with different characteristics and conditions but remained low (Table 6). Implementation practices improved more during the implementation period from a lower baseline (difference-in-difference OR 1.96 (95% CI = 1.47 to 2.63, *P<*0.001).

**Table 6.** Personalised care and support plan comparisons

	Pre-implementation	Post- implementation	Did effect (effect of period in the implementation group over and above any effect of implementation or period alone)			
***Patient cohort***	***n* (%)**	***n* (%)**	**Odds ratio** **(95% CI)**	***P* value**	**Mean difference in percentage** **(95% CI)**	***P* value**
**Implementation practices (*n* = 5060)**	132 (2.6%)	268 (5.3%)				
**Control practices (*n* = 5363)**	711 (13.3%)	790 (14.7%)	1.96 (1.47 to 2.63)	<0.001	1.21 (-4.64 to 7.07)	0.684

Despite this increase in care planning many patients were not aware there had been any care planning during the multimorbidity review (Supplementary Table S4_15):

*'No. No, there was none of that at all. No hint of planning.'* (H42_P24, 75yrs, M)

Many staff understood care plans as condition-specific plans not necessarily given as a record for patients:

*'It depends on what they came in for. Asthma patients [patients with asthma] get an asthma self-management plan ... Diabetics [patients with diabetes] we generally, we discuss and we have a plan … so we do decide on a plan but it’s not necessarily something that we write down for them to take away.'* (H51_P31, nurse, F)

Patients who did receive a care plan felt it was important to have a printed version of this to keep track of their own condition (SupplementaryTable S4 _16).

#### Impact of multimorbidity reviews on patient burden of care

Nurses felt that having fewer annual review appointments for patients could reduce the burden of care for patients (Supplementary Table S4_17) and their carers:

*'… the other day I did a patient with dementia and lots of other conditions and his wife struggles to get him into the surgery said for her it’s much better that, you know, we do as much as we can in that consultation.'* (H32_P11, nurse, F)

Staff felt the longer reviews could be exhausting for patients and therefore staff needed to adapt to this and personalise their information delivery during the review (Supplementary Table S4_18).

## Discussion

### Summary

Following implementation of multimorbidity reviews a greater proportion of eligible patients received a review of their MLTCs in implementation practices, compared with control practices. While the numbers of general consultations in this patient cohort was high, it did not increase during implementation. A before-and-after survey of patients receiving a multimorbidity review showed a small but significant improvement in their overall experience of person-centred care, driven by improved care coordination (see Table 5, *P* = 0.010). A clearer focus on the problems that matter most to patients had benefits for patients’ experiences of care and facilitated a more comprehensive and effective review.

Although the amount of care planning doubled across a broad base of patients in implementation practices, overall, it remained low. Few staff used the care and support plan in the multimorbidity template and tended to think of care plans as being disease specific (for example, asthma plans) rather than holistic. A challenge to offering this holistic person-centred approach was that some of the nurses who conducted these reviews did not feel they were confident or skilled to undertake them.

Overall, the results suggest using the multimorbidity template has potential for greater quality of care and consequently patient benefit with no increase in consultations. The evidence of additional burden on nursing staff does require careful consideration for general practice teams when organising multimorbidity reviews, alongside appropriate training.

### Strengths and limitations

The use of mixed methods provides triangulation and the opportunity to compare and interpret findings by integrating the results. The generalisability of our results is strengthened by our recruitment of practices, both implementation and controls, representing populations from more deprived areas, adding to the research base that often excludes these practices.

The quantitative analysis demonstrated changes in coded, and hence measured, activities, while coding is encouraged by the multimorbidity template, which might explain some of the increases in activities observed in implementation and control practices. To address this, we collected details of a range of codes that might have been used to record similar activities in control practices, rather than just the codes used in the template.

We implemented this study in the first year that practices were required to resume QOF and hence ARs of chronic conditions, following its cessation during the COVID-19 pandemic response. As such, many practices had difficulty with adding a task of sending out questionnaires to patients before their review, which reduced the number of patients who could be sent a follow-up questionnaire, limiting the response rate and hence robustness of this result.

### Comparison with existing literature

We found several positive consequences of using the multimorbidity template in common with previous studies, including providing structure to the consultation, promoting thoroughness and consistency, and facilitating information recording and retrieval.^15^ Improvements in specific person-centred care features were evident for healthcare professionals and patients; for example, having time to ask patients about their priorities and listen to their responses and patients’ experiences of feeling heard, similarly found in other studies.^8^ Little care planning took place as part of this intervention. Providing a template is not sufficient to change staff behaviour. Staff need to be convinced of the value of additional work and its relevance and fit to existing work.^27^ There is a need to re-evaluate the evidence around provision of holistic care and support plans, and if clearly beneficial then accompany this change with staff training at all levels.

### Implications for research and practice

Appropriate staff training is essential to improve care for patients with MLTC. In the UK, most reviews of LTCs are conducted by nurses, rather than doctors, but many nurses do not have the generalist skills necessary to manage a wide range of conditions. GPs are additionally trained in consultation skills, which nurses may lack, but GPs may have become deskilled in some LTCs. The focus of training for nurses has been towards increasing specialisation, but this fails to address the needs of general practice and the growing number of patients with MLTCs, leading to fragmentation and inefficiencies in care. It also underestimates the complex decisions necessary in caring for this patient group. This suggests the need to ensure that nurses in primary care are trained to assess all common conditions, and to reconsider the appropriate contributions of GPs, nurses, and other staff (particularly pharmacists, mental health nurses, and health coaches) in managing patients with complex multiple problems.

Our study did not seek to provide evidence about patient health outcomes. Ideally, this would be collected in a randomised trial, but this is problematic because a shift to integrated, personalised care requires whole-system change, which becomes embedded and normalised in practice, and hence changes in outcomes may not be evident for several years. Additionally, it remains unclear which outcome measures are most appropriate for this patient group.^28^ Future research may need to use long-term quasi-randomised studies based as far as possible on routine data, ideally including routinely collected patient-reported outcomes.

## Trial registration number

This study is registered (ISRCTN 40295449).

## Data

The dataset relied on in this article is not publicly available.
